# Early Life Hormetic Treatments Decrease Irradiation-Induced Oxidative Damage, Increase Longevity, and Enhance Sexual Performance during Old Age in the Caribbean Fruit Fly

**DOI:** 10.1371/journal.pone.0088128

**Published:** 2014-01-31

**Authors:** Giancarlo López-Martínez, Daniel A. Hahn

**Affiliations:** 1 Department of Entomology and Nematology, University of Florida, Gainesville, Florida, United States of America; 2 Department of Biology, New Mexico State University, Las Cruces, New Mexico, United States of America; Leibniz Institute for Age Research - Fritz Lipmann Institute (FLI), Germany

## Abstract

Early life events can have dramatic consequences on performance later in life. Exposure to stressors at a young age affects development, the rate of aging, risk of disease, and overall lifespan. In spite of this, mild stress exposure early in life can have beneficial effects on performance later in life. These positive effects of mild stress are referred to as physiological conditioning hormesis. In our current study we used anoxia conditioning hormesis as a pretreatment to reduce oxidative stress and improve organismal performance, lifespan, and healthspan of Caribbean fruit flies. We used gamma irradiation to induce mild oxidative damage in a low-dose experiment, and massive oxidative damage in a separate high-dose experiment, in pharate adult fruit flies just prior to adult emergence. Irradiation-induced oxidative stress leads to reduced adult emergence, flight ability, mating performance, and lifespan. We used a hormetic approach, one hour of exposure to anoxia plus irradiation in anoxia, to lower post-irradiation oxidative damage. We have previously shown that this anoxic-conditioning treatment elevates total antioxidant capacity and lowers post-irradiation oxidative damage to lipids and proteins. In this study, conditioned flies had lower mortality rates and longer lifespan compared to those irradiated without hormetic conditioning. As a metric of healthspan, we tracked mating both at a young age (10 d) and old age (30 d). We found that anoxia-conditioned male flies were more competitive at young ages when compared to unconditioned irradiation stressed male flies, and that the positive effects of anoxic conditioning hormesis on mating success were even more pronounced in older males. Our data shows that physiological conditioning hormesis at a young age, not only improves immediate metrics of organismal performance (emergence, flight, mating), but the beneficial effects also carry into old age by reducing late life oxidative damage and improving lifespan and healthspan.

## Introduction

Events occurring early in life can have substantial effects on aging, including both mortality rates and healthspan. Numerous studies across a variety of animal species have shown that severe early life stress can cause steeper age-related declines in performance, increased risk of disease and stress-induced pathology, and decreased lifespan [1], [2], [3], [4]. However, in some circumstances early life stress either has no detectable detrimental effects later in life or, particularly if the stress is relatively mild, early life stress can be hormetic and induce changes that leads to greater stress resistance, less age-related loss of performance, and greater longevity [5], [6], [7]. Sub-lethal stress exposure induces physiological changes that can have performance benefits both immediately and later in life, a phenomenon termed physiological conditioning hormesis [4]. Beyond responses to a single stressor, hormetic conditioning with one type of mild stress can induce physiological changes that provide resistance to other types of stressors. For example, hypergravity, heat, cold, and irradiation have been shown to extend longevity in flies [6], and these longevity extensions were accompanied in some instances with increased tolerance to fungal infection.

One of the basic tenets of the free radical theory of aging is that accumulated oxidative damage leads to age-related health and performance declines [8]. These declines are a consequence of an imbalance between reactive oxygen species (ROS), which irreversibly damage membranes, protein, and DNA/RNA and antioxidant defenses [9]. Oxidative damage leads to misfolded or dysfunctional proteins, leaky and fragile cell membranes, and dysfunction in transcription and translation, thus potentially affecting cellular defenses, organismal performance, disease susceptibility, and longevity [10], [11]. Protection against oxidative stress is correlated with longevity, healthspan, and greater resistance to acute or chronic stress in many contexts, including in animals treated with life-extending caloric restriction [12], [13], [14] and careful loss of function and overexpression studies of antioxidant enzymes in some model organisms like *Drosophila melanogaster* flies [15], [16]. Yet, this correlation between low oxidative stress and longevity is not uniform. In recent years both manipulative transgenic studies in laboratory models [17], [18], [19], [20] and studies of long-lived animal species, like naked mole rats and parrots [21], [22], have failed to show a strong relationship between oxidative stress and lifespan. Thus, the field has shifted from a simple view of the free radical theory of aging, wherein oxidative stress is the main driver of senescence, to a more holistic view of the complex process of aging. The recent focus is asking when does oxidative stress and antioxidant protection play an important role in lifespan and healthspan [23], [24]? Because pro-oxidant production is a central component of multiple types of biotic and abiotic stresses, we expect that hormetic treatments enhancing antioxidant defenses may generate cross-tolerance to other stressors that generate oxidative damage. Thus, a mild pro-oxidant stress, could play a critical role in enhancing longevity and healthspan in the face of acute oxidative stress early in life.

Previously we have shown that physiological conditioning hormesis (a short exposure to anoxia) very early in adulthood was successful in increasing total antioxidant capacity and specifically the activity of two antioxidant enzymes, the mitochondrial superoxide dismutase and glutathione peroxidase, for up to 24 hours after anoxia pretreatment in the Caribbean fruit fly, *Anastrepha suspensa* [25]. This adaptive response of boosting antioxidant defenses during a period of low-oxygen availability to prevent extensive oxidative damage upon oxygen reperfusions shares characteristics with a type of hormesis called mitochondrial hormesis (mitohormesis) [26]. Under mitohormesis, increased respiration leads to elevated levels of ROS and the induction of antioxidant defenses. In our model, anoxia lowers and eventually stops respiration, which triggers the adaptive induction of ROS defenses. When treated with a strong oxidative stressor, gamma irradiation, soon after experiencing anoxic conditioning, male flies receiving the hormetic treatment had lower oxidative damage and substantially better performance in treatment survival, emergence, flight, and mating [25]. In the context of mating, the sexual performance of young male flies was enhanced in anoxic preconditioned individuals 12 days after the irradiation stress, even though elevated levels of antioxidant activity induced by anoxic preconditioning only lasted for 24 hours.

In the present study, we explored the relationship between antioxidant-mediated hormesis, and longevity and healthspan by testing the potential of a hormetic treatment, anoxic preconditioning, delivered just before and during an intense early life exposure to oxidative stress, gamma irradiation, to extend lifespan and healthspan. In one experiment, we exposed flies to low non-lethal doses of ionizing radiation known to induce sterility and substantial oxidative stress. In a second experiment, we exposed them to higher doses known to generate severe oxidative stress, precipitously decrease organismal performance early in life, and lead to premature death. In addition to tracking longevity, we compared male mating success at young and old ages as a metric of healthspan and quantified metrics of oxidative damage at young and old ages. We provide evidence that physiological conditioning hormesis (i.e. a short exposure to anoxia that transiently increases antioxidant defenses) leads to an increase in lifespan and healthspan in fruit flies and that avoidance of excess oxidative damage is associated with increased sexual performance at old age.

## Materials and Methods

### Animal rearing

Caribbean fruit flies, *Anastrepha suspensa* (Loew) (hereafter caribflies) were taken from a colony reared at the Florida Department of Agriculture and Consumer Services (FDACS) in Gainesville, Florida. We chose caribflies because they are mass-reared at this facility and used in an environmentally-friendly control tactic known as the sterile insect technique (SIT). For SIT males are sterilized with ionizing radiation and released into infested areas to lower the population by mating with wild females [27]. These sterilized males suffer from poor performance due to reactive oxygen species formation during irradiation. We have previously shown that anoxia-conditioning hormesis decreases post-irradiation oxidative stress and improves post-irradiation mating performance in young flies in this system [25]. Larvae and pupae were maintained on damp vermiculite in an incubator (Percival Scientific, Perry, Iowa, USA) at 24°C and 85% RH under long day conditions (14L∶10D). Prior to emergence, pupae were transferred to standard 30 cm×30 cm×30 cm screened insect cages and kept in a temperature (25°C) and humidity (60% RH) controlled rearing room with access to food (3sugar:1yeast hydrolysate paste), and water *ad libitum*.

### Irradiation treatments

Pharate adult flies, still inside the puparium, were irradiated two days prior to emergence using a Gammacell Cs^137^ irradiator (GC45, Ottawa, ON, Canada) at a dose rate of 8.948 Gy/min at the Florida Accelerator Services and Technology facility within the Division of Plant Industry of FDACS. Pupae were confined to polypropylene bags and placed in the center of the irradiation cylinder to ensure dose uniformity. Gafchromic HD-810 film (International Specialty Products, Wayne, NJ, USA) was used to verify the accuracy of the target dose by placing three dosimeters per bag (top, middle, and bottom) and reading them 24 hrs after irradiation. Actual delivered irradiation doses were within 10% of target doses. Irradiation was performed under one of two atmospheric treatments: in the presence of oxygen (normoxia-nx) or in the absence of oxygen after an hour-long anoxia conditioning pre-treatment in a nitrogen atmosphere (anoxia-ax). Anoxia prior to and during irradiation is our hormetic treatment that leads to enhanced antioxidant capacity and lower post-irradiation oxidative stress [25]. Irradiation treatments for our two experiments included the following doses (exposure times): experiment 1 compared a low-dose of 70 Gy (7 min and 49 sec) with 0 Gy, and experiment 2 (high-dose experiment) compared 0 Gy with 200 Gy (22 min and 21 sec), 300 Gy (33 min and 32 sec), and 400 Gy (44 min and 42 sec). 70 Gy is the target dose that leads to 100% sterility under both normoxic and anoxic conditions in caribflies but does not prevent adult emergence, strongly impact flight performance, or induce early-life mortality [28], [25]; making 70 Gy an appropriate target for our first experiment. In the second experiment, to evaluate whether the same anoxia pre-treatment could have a benefit at higher doses; 200 and 300 Gy were chosen because they should generate greater oxidative damage and possibly greater declines in post-irradiation performance and longevity. 400 Gy was our highest dose, known to induce severe damage and immediate high mortality [25].

Our physiological conditioning hormesis treatment, anoxia, was implemented by placing pupae in airtight polypropylene bags that were flushed with nitrogen and heat sealed, as previously described [25]. Pupae in the normoxia treatments were sealed in similar bags that had been thoroughly perforated to allow air exchange. To ensure treatment uniformity in the low-dose experiment (70 Gy), anoxia non-irradiated control pupae (AxNr) were kept eight additional minutes in anoxia to receive the same length of anoxia exposure as the anoxia irradiated (Ax70) pupae. For the high-dose experiment, the anoxia no radiation (AxNr), 200 Gy (Ax200) and 300 Gy (Ax300) groups had their anoxia exposures adjusted by 44, 22, and 11 minutes, respectively, to standardize the duration of the anoxic conditioning to that experienced by the 400 Gy (Ax400) treatment group (1 hr and 44 minutes). Anoxia-irradiated individuals were not reperfused with normoxic air until after irradiation had ended.

### Longevity

After conditioning and irradiation treatments, groups of pharate adult flies were allowed to emerge inside wire-screen cages (30 cm×30 cm×30 cm) and their survival was tracked on a weekly basis. For both the low-dose and the high-dose experiments, three replicate cages of 200 pharate adults were set for each treatment (specifically for the low-dose experiment: normoxia no radiation, anoxia no radiation, normoxia 70 Gy, and anoxia 70 Gy; and for the high-dose experiment: normoxia no radiation, anoxia no radiation, normoxia 200 Gy, anoxia 200 Gy, normoxia 300 Gy, anoxia 300 Gy, normoxia 400 Gy, anoxia 400 Gy). Once a week, dead flies were removed from the cages, sexed, and counted. Food (sugar and yeast hydrolysate paste) and water were replaced weekly to ensure a surplus of both was present. In each cage, flies were also provided a substrate for oviposition using yellow-dyed 2% agar domes wrapped in parafilm [25], [29]. However, irradiated flies never laid eggs in these domes during these experiments. In the low-dose experiment we did not carry out the longevity trial until all flies died. Rather the remaining living flies were counted for rate of adult emergence, sexed, and survival was determined at 10 weeks (their natural lifespan in the field, [30]). For the high-dose experiment we followed all individuals until they died (22 weeks). Data are presented as means and standard errors.

### Sexual performance of young and old flies

**Figure 1:**
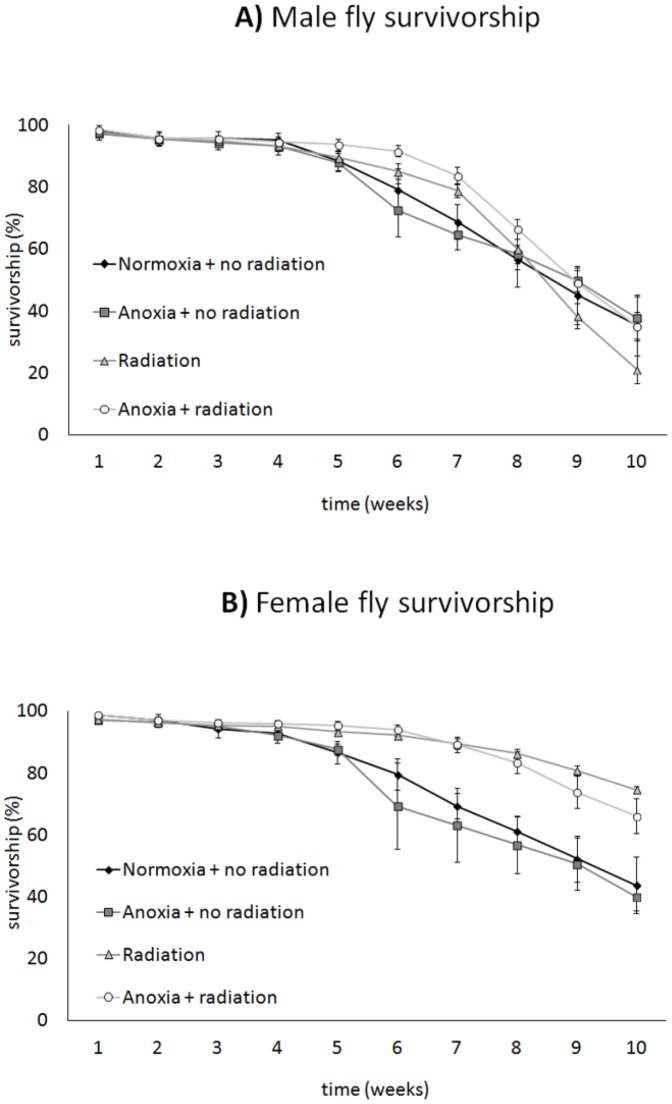
Male and female longevity was tracked for ten weeks in the low-dose experiment by placing 200 flies prior to adult emergence in replicate insect cages. Mortality rates were reduced in both sexes by sterilizing ionizing radiation. Hormetic conditioning only had a lifespan extension effect in males (**A**). Female longevity (**B**) was extended by irradiation but not further extended by hormetic conditioning. Means and standard errors across replicate cages are plotted in the graph.

Pupae were dusted with orange, blue, or green fluorescent powder (DayGlo, Cleveland, OH, USA) prior to adult emergence. This powder allowed the identification of treated males without affecting fly performance or female mate choice [25], [31]. Virgin irradiated and unirradiated males were separated and isolated in their own cages within five days of emergence, before they became sexually competent, and maintained as described earlier [25]. Our previous work showed that anoxia had hormetic effects on irradiated flies that strongly increased sexual performance 10 days after emergence (12 days after treatment) and thus we investigated whether this benefit was still prevalent weeks after treatment. Based on the mortality differences between the 4^th^ and 5^th^ weeks (Fig. 1), where strong differences arise at the 70 Gy level, we used 30 days as our time point for old flies. Choice test mating trials were performed at either young (10 days after emergence) or old (30 days after emergence) ages using the same cohort of flies for both time points. For the old age trial, we used 30 day old virgin males and young (10 days old) untreated (normoxia and no radiation) virgin females. Two males from different treatments (NxNr vs. Nx70, NxNr vs. Ax70, or Nx70 vs. Ax70) were placed in 8.5 cm high and 7 cm diameter wire mesh cages and allowed to acclimate for 20 minutes. An untreated virgin female was then added to the cage and the outcome (successful male and time to mating) was recorded. In some trials, females were unresponsive and did not initiate courtship with either male after one hour of active courting by males. These unresponsive females were removed and replaced with fresh virgin females. For both 10 day (young male) and 30 day (old male) competitive trials, six replicate groups of 15 individual pairings were used. Two groups within each of three cohorts were performed for the two most salient comparisons: NxNr vs. Ax70 and Nx70 vs. Ax70. These trials were performed over three weeks, using different fly cohorts each week. For the old male versus young male competitive trial, old (30 day) anoxia-irradiated (Ax70) flies were competed against young (10 day) normoxia-irradiated flies (Nx70) from different cohorts. Data are presented as the average percentage of successful mating events.

### Total antioxidant capacity

After mating trials, flies were collected, frozen in liquid nitrogen, and stored at −70°C until assayed. Antioxidant capacity was estimated using the ABTS radical cation decolorization assay to measure trolox-equivalent antioxidant capacity (TEAC; [32]). Three replicate pools of 5 pupae each (∼50 mg) per treatment were homogenized in PBS using a Fast Prep 120 bead homogenizer (Qbiogene Inc., Carlsbad, CA, USA) with 2 mm zirconia beads (Biospec Products Inc.). After homogenization and protein quantification at 260 nm using a NanoDrop 1000 spectrophotometer (Thermo Scientific, Wilmington, DE, USA), homogenates were diluted to a concentration of 2 mg of protein/ml. A solution of 7 mM ABTS (2, 2′-Azino-bis-(3-ethylbenzothiazoline-6-sulfonic acid)) and 2.45 mM potassium persulfate (Sigma-Aldrich, St. Louis, MO, USA) that had been incubated overnight was added to the samples and 10 minutes later the absorbance of the samples was read at 734 nm. Samples were quantified using an eight point Trolox standard curve (0–150 µM/ml) and data are expressed as Trolox equivalents per mg of soluble protein. We have previously shown that an increase in total antioxidant capacity is accompanied by increases in the enzymatic activities of the mitochondrial Superoxide dismutase and Glutathione peroxidase [25], but here we only assayed TEAC.

### Oxidative damage

Lipid peroxidation was estimated using the thiobarbituric acid reactive substances test (TBARS), adapted from one previously described [33], [34]. Pools of five male flies (∼50 mg) were homogenized as described above, but in radioimmunoprecipitation assay buffer (RIPA) buffer containing EDTA (Fisher Scientific, Fair Lawn, New Jersey, USA). The homogenates were separated into aliquots for protein quantification and oxidative damage determination respectively. After treatment with 10% trichloroacetic acid to precipitate out the proteins, the homogenate was combined with a 0.67% (w/v) thiobarbituric acid solution and heated (95°C) for one hour. Cooled samples were centrifuged (2,200 g for 5 min at 25°C) and dispensed into a 96-well plate. Sample absorbance was read at 532 nm and MDA levels were quantified using an eight point malondialdehyde (MDA) standard curve (0–50 µM/ml). Data are presented as µM of MDA per mg of soluble protein.

Protein oxidation was estimated using the 2, 4-dinitrophenylhydrazine (DNPH) method [35]. After homogenization in a 5% sulfosalicylic acid solution, samples were treated with DNPH or HCl and incubated for one hour with occasional centrifugation. Samples were incubated on ice after the addition of trichloroacetic acid, followed by repeated washes in a 1∶1 ethanol∶ethyl acetate solution to extract the remaining excess DNPH. The final protein pellet was eluted in 6 M guanidine hydrochloride. Samples were then read at 370 nm against sample blanks processed in HCl. The protein blanks and a 9-point BSA standard curve (0–2 mg/ml) were used to quantify protein concentration and standardize the results to 1 mg of protein. Data are presented as nmol per mg of soluble protein.

### Statistical analyses

Each of the longevity experiments was performed two separate times using different cohorts of flies. The mating trials were conducted with 6 replicates across three weeks using different fly cohorts but 10 day (young fly) and 30 day (old fly) trials were carried out within each cohort. Longevity data were analyzed using a proportional hazards model with anoxia treatment, irradiation dose, sex, and their interactions as variables. A log-rank test was additionally used to analyze the low-dose experiment due to the fact that it contained censored data (the experiment was terminated before 100% mortality was reached). Mating data were analyzed using logistic regression. Total antioxidant capacity, TBARS, and protein carbonyls were analyzed with two-way ANOVAs followed by Tukey's HSD or linear contrast tests to directly compare groups of interest.

## Results

### Longevity

Both anoxia hormesis and irradiation affected longevity in caribflies in the low-dose experiment (70 Gy vs. 0 Gy) (Fig. 1; p_model_ = 0.0003, p_oxia treatment_ = 0.0275, p_radiation_ = 0.0002, p_sex_ = 0.0073). When we examined the sexes separately, longevity extension by anoxic-conditioning hormesis was only detectable in males (Fig. 1; Table 1). Hormesis conditioning caused a shift in the survivorship curve of males and the difference in survivorship between the hormetic-irradiated and the irradiated males increased over time (log-rank *X* ^2^ = 26.38, p<0.0001). In contrast, female longevity was only extended by irradiation and not additionally extended by our hormetic treatment, with sterilized females showing nearly 50% higher survivorship at week 10 than fertile females regardless of anoxia hormesis treatment (log-rank *X* ^2^ = 9.47, p = 0.0236).

**Table 1:** Proportional hazard models were used to analyze mortality rate and longevity in both the low-dose (**A**; 0 and 70 Gy) and high-dose (**B**; 0, 200, 300, 400 Gy) experiments.

A) Low dose experiment									
treatment	χ^2^	d.f.	p_model_	p_oxia treat_	p_radiation_	p_sex_	p_oxia×radiation_	p_oxia×sex_	p_oxia×radition×sex_
full model	23.35	5	0.0003*	0.0275*	0.0002*	0.0073*	0.8037	0.4404	0.5824
separate models									
70 Gy males	16.41	3	0.0009*	0.0231*	0.0004*	N/A	0.466	N/A	N/A
70 Gy females	5.94	3	0.1147	0.2387	0.0435*	N/A	0.783	N/A	N/A

B) High dose experiment									
treatment	χ^2^	d.f.	p_model_	p_oxia treat_	p_radiation_	p_sex_	p_oxia×radiation_	p_oxia×sex_	p_oxia×radition×sex_
full model	4550.32	15	<0.0001*	<0.0001*	<0.0001*	<0.0001*	<0.0001*	<0.0001*	<0.0001*
separate models									
0Gy-high dose	45.67	3	<0.0001*	0.039*	N/A	<0.0001*	N/A	0.3839	N/A
200Gy-high dose	823.25	3	<0.0001*	<0.0001*	N/A	<0.0001*	N/A	<0.0001*	N/A
300Gy-high dose	1018.55	3	<0.0001*	<0.0001*	N/A	<0.0001*	N/A	0.0108*	N/A
400Gy-high dose	752.81	3	<0.0001*	<0.0001*	N/A	<0.0001*	N/A	<0.0001*	N/A

Here we include the complete analysis showing the whole model and separate models by sex (**A**) or irradiation dose (**B**). Significant values at the 0.05 level are marked by asterisks (*).

**Figure 2:**
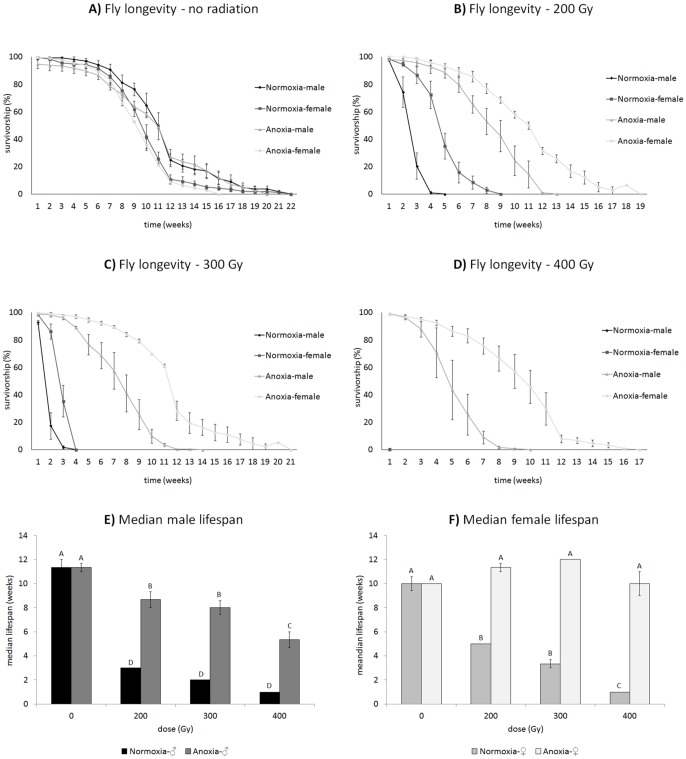
For the high-dose experiment, longevity was recorded for both sexes in response to four irradiation doses (0, 200, 300, and 400 Gy) and either with or without hormetic conditioning (anoxic vs. normoxic) after placing 200 flies prior to adult emergence in replicate insect cages and tracking them until all individuals had died. There was a slight negative effect of anoxia conditioning in the absence of irradiation (**A**), which increased the mortality hazard rate. However, anoxic-conditioned and irradiated flies had lower mortality rates and increased lifespan compared to both unirradiated and unconditioned flies of both sexes (**B–D**). This lifespan extension effect was more pronounced in females; a possible interaction of conditioning and sterility ameliorating the costs of reproduction. **E**) A multivariable ANOVA of the median lifespan of males in (**A–D**) shows an increased in lifespan by anoxia-conditioning prior to irradiation but a reduction by irradiation in oxygen (F_7,16_ = 75.5714; p_model_<0.0001, p_oxia treatment_<0.0001, p_dose_<0.0001, p_oxia treatment * dose_<0.0001). **F**) Female median lifespan shows a similar patterns to males but with a more robust hormetic effect (F_7,16_ = 87.1837; p_model_<0.0001, p_oxia treatment_<0.0001, p_dose_<0.0001, p_oxia treatment * dose_<0.0001). Means and standard errors across replicate cages are plotted in the graphs. Groupings on **E** and **F** are based on a Tukey's HSD correlation for multiple comparisons.

Anoxia hormesis, irradiation, and sex, as well as their interactions had strong effects on longevity in the high-dose experiment (Figs. 2; Table 1). When we separately analyzed the treatments by irradiation dose, anoxia conditioning had a slightly deleterious effect on non-irradiated flies (Fig. 2A; Table 1). Female longevity was extended by irradiation at all doses (200, 300, and 400 Gy; Fig. 2B–D). Anoxia-conditioned and irradiated females had dramatically longer median lifespan than anoxia-conditioned and irradiated males (Fig. E–F; F_15,32_ = 78.4711; p_model_<0.0001, p_oxia treatment_<0.0001, p_dose_<0.0001, p_sex_<0.0001, p_oxia treatment * dose_<0.0001, p_sex * dose_<0.0001, p_oxia treatment * sex_ = 0.0001, p_oxia treatment * dose * sex_ = 0.0043), a likely interaction of sterility eliminating the high costs of reproduction on longevity in females and lower somatic damage. Thus both hormetic-irradiated males and females benefit from the anoxia conditioning at high doses. At 200 Gy, both male and female longevity in the hormetic-treated group was more than double that of flies that were irradiated without anoxia treatment. At 300 Gy, lifespan extension in the hormetic flies ranged from 3 times longer in males to more than 5 times longer lifespan in females compared their irradiated counterparts that did not receive the hormetic conditioning treatment. Flies in the 400 Gy group that were irradiated without conditioning did not survive more than a day or two, but flies that were irradiated at 400 Gy in anoxia after hormetic conditioning lived substantially longer with males living up to 10 weeks and females 17 weeks.

### Sexual performance

**Figure 3:**
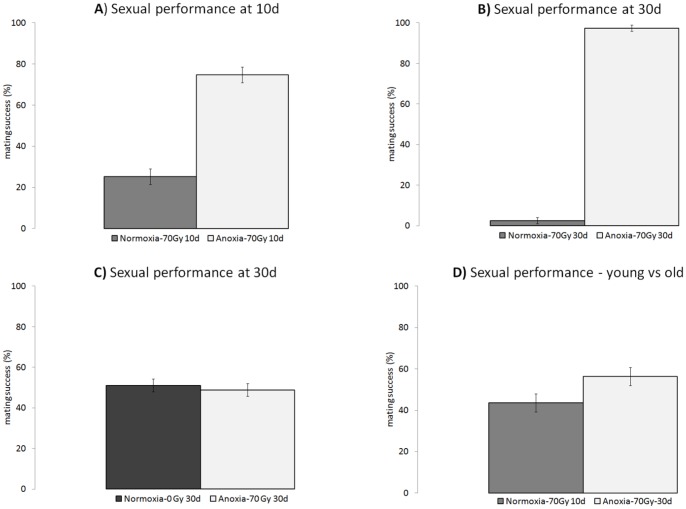
Mating success was studied at young (10 days) and old (30 days) age by performing competitive mating trials using two differently-treated males and an untreated female. Young hormetic-irradiated males (**A**) were more successful at mating than young unconditioned-irradiated males. At old age, hormetic-irradiated males had even higher mating success compared to unconditioned-irradiated males (**B**), but were equally as sexually competent as old unirradiated males (**C**). Furthermore, old hormetic-irradiated males mated with the same frequency as young unconditioned irradiated males (**D**). Means and standard errors across replicate trials are plotted in the graph.

Young (10 d) anoxia-conditioned irradiated males outperformed young males that did not receive anoxia hormesis treatment prior to irradiation in competitive mating trials (χ^2^ = 41.8599, p<0.0001; Fig. 3A), as had been previously shown [25]. Later in life (30 d), male flies that had received the anoxia hormetic treatment prior to irradiation had even better sexual performance compared to old males irradiated without anoxia hormesis, with almost 100% of successful mating events going to anoxia-conditioned males (χ^2^ = 149.7917, p<0.0001; Fig. 3B). We competed old anoxia-conditioned and irradiated males against old males that had not been irradiated and mating success was nearly identical (χ^2^ = 0.039218, p = 0.9998; Fig. 3C); showing strongly that our anoxic-conditioning treatment rescued the deleterious effects of irradiation on the mating performance of old males. Because anoxia conditioning appeared to preserve mating performance so well, we also competed old (30 d) anoxia-conditioned and irradiated flies against young (10 d) irradiated flies that had not received anoxia conditioning. Old (30 d) anoxia-conditioned and irradiated males were equally successful at mating as young (10 d) unconditioned and irradiated males (χ^2^ = 2.05, p = 0.1517; Fig. 3D).

### Total antioxidant capacity and oxidative damage

**Figure 4:**
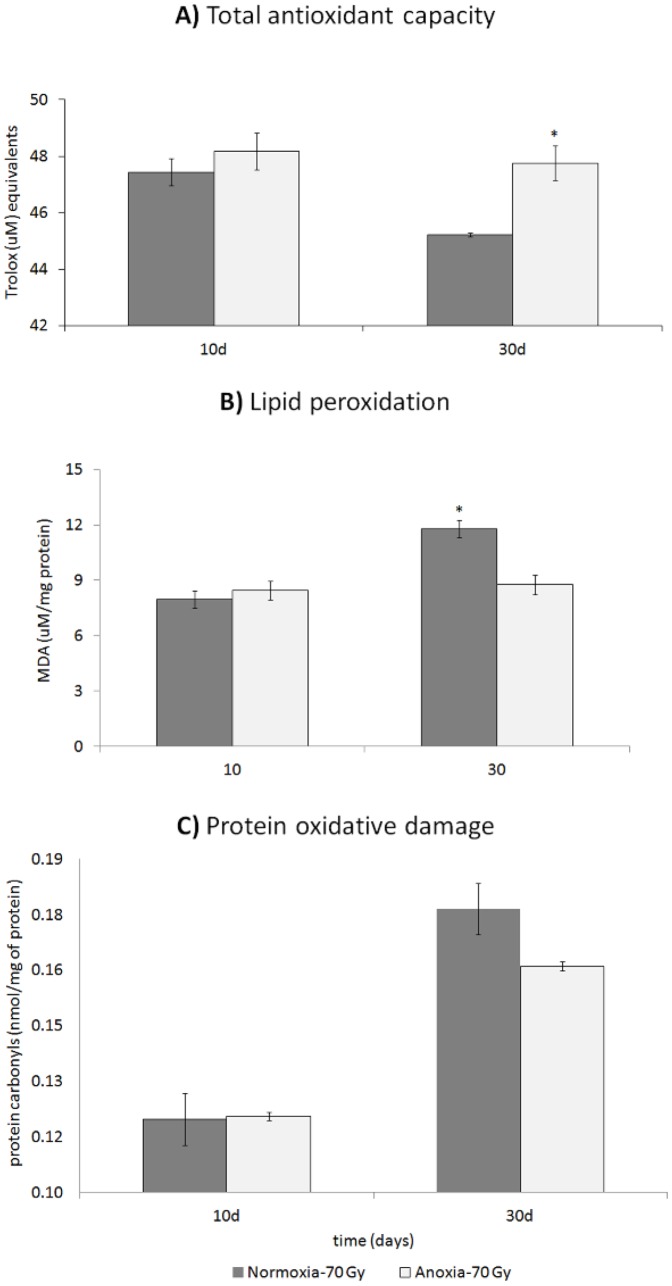
We assessed total antioxidant capacity and two markers of oxidative damage (lipid peroxidation and protein carbonyls) in male flies following the end of the mating trails. Total antioxidant capacity (**A**) did not differ between young males after mating (10 days after hormesis). At old age however, hormetic-irradiated flies had higher antioxidant capacity (30 days after hormesis). Oxidative damage to lipids (**B**) did not differ in young flies but was lower in old hormetic-irradiated flies. Protein oxidation damage (**C**) was overall higher at old age but there were no treatment differences. Asterisks denote significant differences from linear contrasts comparing hormetic-treated and non-hormetic flies. Means and standard errors are plotted in the graph.

There was a clear increase in antioxidant capacity in the anoxia-irradiated males flies at old age (30 d), but there was not a statistically detectable change in antioxidant capacity in young male flies (10 d) (F_3,5_ = 4.7239; p_model_ = 0.0638, linear contrast p = 0.032; Fig. 4A). Similarly, at old age (30 d), anoxia-irradiated male flies had lower oxidative damage to their lipids (F_3,7_ = 4.7051; p_model_ = 0.042, p_oxia treatment_ = 0.629, p_day_ = 0.0248, p_oxia treatment * day_ = 0.046, linear contrast 0.0266; Fig. 4B), but there was no detectable effect of anoxia conditioning on lipid peroxidation in young males. There was no effect of anoxic conditioning on protein oxidation after mating either at young or old age, but older flies had more damage than younger flies regardless of conditioning (F_3,8_ = 4.9413; p_model_ = 0.0315, p_oxia treatment_ = 0.9718, p_day_ = 0.0056, p_oxia treatment * day_ = 0.545, linear contrast 0.0056; Fig. 4C).

## Discussion

Previously we had shown that our hormetic treatment (an hour-long bout of anoxic conditioning) applied during the early adult stage induced an increase in total antioxidant capacity [25]. This increase in antioxidant capacity was associated with strong elevations in the enzyme activities of MnSOD and GPx. Furthermore, when our anoxic-conditioning treatment was combined with irradiation in anoxia, flies had less damage to lipids and proteins and greater post-irradiation performance (i.e. emergence, flight, and mating). Here we report that the benefits of anoxia conditioning hormesis during early adulthood extend far into adulthood and these benefits are even more pronounced at old age. Male flies irradiated after anoxic conditioning showed a significant decrease in the hazard mortality rate and an increase in longevity across all irradiation treatments considered here. In addition to living longer, these hormetic-irradiated male flies were more successful at mating later in life than their unconditioned-irradiated counterparts. Female flies lived significantly longer after irradiation compared to non-irradiated females. After irradiation, female ovaries do not continue to develop, they atrophy, and eggs present at that time are potentially reabsorbed [36], [37]. Because all doses used in our experiment blocked female reproduction, the investment these females would normally allocate for reproduction could be distributed to lifespan extension, as has been observed in a wide range of taxa from insects to lizards [38], [39], [40], [41], [42].

In the low-dose experiment, the effect of anoxic conditioning was male-specific, we detected no benefit of anoxic conditioning with irradiation in females at the 70 Gy dose. Maximum lifespan for these flies in the lab is about 21 weeks and exposure to anoxia by itself shortens female lifespan by 25%. At higher doses of irradiation, anoxic conditioning hormesis was shown to have dramatic beneficial effects on treatment survival, adult emergence, and flight ability [25]. When considering our higher doses of irradiation, males were more radiation sensitive than females and most males were dead by 3 weeks after exposure to 200 Gy, 2 weeks after exposure to 300 Gy, and just a day or two days after exposure to 400 Gy. Consistent with our observations in the low-dose experiment at 70 Gy, females did not reproduce and lived longer than males at all three higher doses. Anoxic-conditioning hormesis triggered a shift in the mortality curve at the 200 and 300 Gy doses and increased maximum longevity of the treated population by two or three times in the case of males with even more pronounced lifespan extensions in females. Although we did not quantify activity, anecdotal observations suggest that increases in longevity in the anoxic-conditioning groups were accompanied by increased organismal activity compared to the unconditioned-irradiated individuals. Specifically, we observed qualitative differences in behavior and performance where unconditioned-irradiated flies were not able to fly normally, walk quickly, or right themselves after falling on their backs and appearing dead, occasionally flying in circles while remaining upside-down temporarily and eventually righting themselves. These *degenerative behaviors* have been previously identified as being related to lifespan [43]. The supine behavior (temporary upside-down orientation) was first described in another fruit fly, *Ceratitis capitata* [44], and is a behavior normally displayed during advanced age nearing death [43]. We observed that supine behavior was more frequent in treatments that suffered increased oxidative damage to lipids and proteins and was less frequent in anoxia-conditioned treatments that had less oxidative damage late in life. It is possible that the onset of age-related supine behavior is linked to increased oxidative damage in crucial tissues like thoracic flight muscles. Anoxic-conditioned flies continued to remain physically active and sexually competitive after irradiation late in life when their unconditioned-irradiated counterparts were experiencing severe behavioral and motor-skill dysfunction.

Lifespan extension receives much attention; however, emphasis is now shifting from absolute lifespan to the concept of healthspan, wherein performance at old age is an important metric of successful interventions [23], [24]. Here we assess male mating performance at young (10 days) and old age (30 days) as our metric of healthspan. Mating is a costly process for any organism [45], and this cost can be divided into production of gametes, sexual display, and copulation. The complexity and expenditure of the mating process is very well understood in insects, particularly fruit flies [45], but the effects of mating and stress on healthspan are less well studied. In our work, female caribflies preferred irradiated males that received hormetic conditioning over unconditioned-irradiated males 3 to 1 when young, but females preferred old hormetic-conditioned irradiated males over old unconditioned irradiated males 19 to 1. Thus the effects of hormetic conditioning on healthspan are well illustrated by the very clear preferences for old- conditioned males when exposed to irradiation stress. The fact that unconditioned irradiated male flies had more oxidative damage in lipids and lower antioxidant capacity at old age suggests that females may prefer old hormetic flies because they have less damage and may be more active due to less degenerative behaviors (i.e. supine). Females showed no preference when offered a choice between old-unirradiated and old hormetic-irradiated males. Additionally, old hormetic-irradiated flies share the same mating success as young unconditioned-irradiated ones, even though the anoxic-conditioned males were three times as old, females did not discriminate between them and the young irradiated ones. Thus, hormesis while lowering oxidative damage and increasing healthspan also prevents the onset of age-related degenerative behaviors.

It is clear that the strong effects on organismal performance we attribute to hormesis could arise from multiple biochemical pathways and antioxidant enzymes are probably not solely responsible for this effect. The hypoxia-reperfusion response (similar to mitohormesis), wherein an organism increases cellular defenses during periods of low oxygen in preparation for metabolic resumption, is known to increase other non-enzymatic antioxidant compounds like glutathione and glutathione disulfide [14], [46], chaperones like heat shock proteins (Hsps; [47]), and the activation of numerous signal transduction pathways [48]. Other biochemical players like trehalose are known to be critical at preventing protein aggregation during long periods of anoxia in yeast, *Drosophila*, and mammalian cells [49], [50], [51] and to extend longevity in *C. elegans* [52]. Additionally molecular chaperones like hsps are involved during periods of anoxia [53], [54], [55], reperfusion [47], and irradiation [56]. Hsps and their reduction in protein denaturing and aggregation are even linked to aging [57]. Additional work is needed to describe the multifarious biochemical and cellular effects of our anoxia-conditioning treatments in caribflies, and how inducing these cellular changes leads to hormetic cross-tolerance for irradiation stress.

In summary, we showed that at both sub-lethal and acute-lethal irradiation doses, anoxia-conditioning hormesis led to significant increases in lifespan and performance in both males and females. Females showed a robust increase in lifespan after treatment that likely represents an interaction between hormesis and reduced reproduction due to irradiation sterilization. The lifespan extending effects of anoxic-conditioning hormesis at our sub-lethal dose (70 Gy) were also correlated with increases in mating success at old age, our metric of healthspan. Even though anoxia irradiated males were still sterile, they outcompeted unconditioned-irradiated males in head-to-head mating trials, and we believe that the somatic protection gained from anoxic conditioning preserves their sexual competitiveness making them as successful as non-irradiated males. The robust increase in mating performance at old age was correlated with higher antioxidant capacity and lower oxidative damage at old age, a full month after the anoxic-conditioning and irradiation treatment. Our data indicates that an anoxia-induced boost in antioxidant defenses before an exposure to a strong oxidizing stressor during young age has hormetic effects that extend into adulthood through old age, ameliorating both mortality rates and reproductive senescence.

Beyond basic research on lifespan and healthspan, these results have implications for direct applications to the Sterile Insect Technique (SIT) in insect pests. SIT is an environmentally friendly, area-wide pest control tactic that has been effectively used as part of integrated pest management strategies for tephritid fruit flies (including the Caribbean fruit fly *A. suspensa* – used here, the Mexican fruit fly *A. ludens*, the Mediterranean fruit fly *Ceratitis capitata*, and more), moths, tsetse flies, and mosquitoes [58]. In SIT programs, male insects are typically irradiation-sterilized and sent out into the field to disrupt pest populations by mating with wild females. Even though irradiation very effectively sterilizes insects, irradiation is known to have substantial negative side effects on performance. Here we have shown that a simple anoxic-hormetic treatment can improve both the longevity and old-age mating success of irradiated males. Several authors before us have shown that anoxic-conditioning and irradiation in anoxia can improve early-life performance [59], [60], [61]. To our knowledge, ours is the first study to investigate effects of anoxic-conditioning treatment on healthspan and oxidative stress, showing that both oxidative damage associated with irradiation is reduced in old males and that conditioning improves the sexual performance of old males. We hope that our results will motivate others to explore simple hormetic treatments to increase the healthspan of irradiated insects so conditioning treatments can ultimately be used to improve the efficacy and affordability of environmentally friendly, non-pesticidal SIT programs.
